# Diversifying Methods in Evolutionary Anthropology: Autophotography as a Tool for Quasi‐Naturalistic Observation of Human Behavior

**DOI:** 10.1002/evan.70037

**Published:** 2026-07-01

**Authors:** Emily H. Emmott, Masahito Morita

**Affiliations:** ^1^ UCL Anthropology University College London London UK; ^2^ Faculty of Economics Shiga University Shiga Japan; ^3^ Department of Biological Sciences The University of Tokyo Bunkyo Japan; ^4^ Faculty of Humanities Nanzan University Nagoya Japan; ^5^ Department of Health Sciences of Mind and Body University of Human Arts and Sciences Iwatsuki Japan

**Keywords:** autophotography, human behavior, observational method, qualitative methods

## Abstract

A comprehensive evolutionary account of human behavior demands simultaneous consideration of biology, local ecology, and culture. Historically, evolutionary anthropology integrated these dimensions through naturalistic observations which offered direct insight into how behaviors unfold within everyday settings. However, in many contemporary contexts, traditional observations are impractical due to challenges in accessing private spaces, contributing to the decline of such methods. While evolutionary anthropologists have gradually pivoted to experimentation and larger datasets, the contextual richness afforded by direct observation is not readily replaced by other approaches. In response, and in light of renewed interest in investigating mechanisms and methodological diversification, we introduce and reflect on *autophotography*—a visual method in which participants document aspects of their lives through photographs—as a pragmatic, quasi‑naturalistic observational tool for evolutionary anthropology. Within its limitations, this creative, participant‑led approach may help recover some strengths of naturalistic observation while adapting to contemporary field sites.

1

Ethologist Nikolaas Tinbergen proposed four questions researchers must address to understand behaviors: what are the biological mechanisms that cause the behavior; how does the behavior develop; what is the adaptive function of the behavior; and how did the behavior evolve [1]. These questions provided tools to address behavioral complexity, becoming highly influential within evolutionary anthropology and beyond [2]. Unlike (most) other animals, however, applying Tinbergen's questions to human behavior also require understanding cultural complexities: behavioral mechanisms and development are rarely purely biological, and human evolution cannot be understood without cultural evolution. Thus, a comprehensive investigation of human behavior must simultaneously consider biology, local ecology, and culture.

Historically, evolutionary anthropology drew heavily on naturalistic, in situ observational methods, allowing researchers to examine behavior as it unfolded within everyday ecological and social contexts [1, 2, 3]. This tradition, rooted in ethology and behavioral ecology, provided direct insight into how behavior was shaped by local ecological conditions. Human behavioral ecology in particular has long examined how local ecologies influence fitness‐related behaviors through sustained, context‑rich observation [4, 5]. The value of such observational methods was recognized early on: in *The Expression of Emotions in Man and Animals*, Charles Darwin himself emphasized the value of naturalistic observations because, “In observing animals, we are not so likely to be biassed by our imagination” ([6]; p. 17). Similarly, Tinbergen warned researchers against forgoing naturalistic observations to avoid “losing touch with the natural phenomena” ([1]; p. 411).

Despite this strong observational foundation, naturalistic observation as a method has declined within evolutionary anthropology. Practical and ethical constraints—particularly in post‑industrialized contexts where much of daily life occurs in private or inaccessible spaces—can make direct observations difficult. At the same time, contemporary research culture increasingly prioritizes experimental designs and large sample sizes for advanced statistical modeling, creating fewer incentives for the slow, labor‑intensive, and context‑rich data that naturalistic observation produces. As a result, one of the few methods that offered direct insight into behavior as it unfolds in natural settings ‐and the depth of contextual knowledge that comes with such observation‐ has become increasingly difficult to sustain. This decline matters: without ways to observe behavior in situ, opportunities to integrate biological, ecological, and cultural perspectives are diminished. Direct observations allow researchers to see how cues, constraints, and cultural expectations jointly shape behavior as it unfolds, rather than inferring these relationships indirectly. There is therefore a growing need for alternative approaches that can approximate the strengths of naturalistic observation while adapting to modern research settings.

## 2 A Case for the Return of Naturalistic Observations?

Recently, several evolutionary anthropologists have stressed the importance of investigating underlying mechanisms alongside testing evolutionary hypotheses [7, 8], and has highlighted the value of methodological diversification [8, 9]. Naturalistic participant observation is a skill the discipline already possesses [3], and one that enables researchers to trace the proximate processes through which behavior unfolds in situ, shaped by local norms and ecologies.

The epistemic value of naturalistic observation also deserves emphasis. While its methodological lineage is rooted in ethology, contemporary reflexive approaches highlight that researchers’ understandings of behavior are inevitably shaped by subjective and culture‑specific assumptions. Observing behavior in everyday settings can therefore act as a check on these assumptions, reducing interpretive bias, and enabling more context‑sensitive and objective accounts of human behavioral variation [3, 10].

However, as noted above, conducting naturalistic observations in post‑industrialized settings often presents practical and ethical challenges. Much of everyday life occurs in private or semi‑private spaces (e.g., homes, workplaces, schools) that are inaccessible to researchers, and even when access is granted, researcher presence may be so unusual that it amplifies observer effects. In our own work, we have experimented with various methods including combining self‐reported behavioral data with focus groups to facilitate interpretation [11], or using webcam footage to capture dyadic interactions without observer effects [12, see also 13]. Yet these approaches still provide only partial insight: focus groups rely on self‐report primarily, while webcam observations capture interactions but not the broader ecological or social contexts in which behavior occurs.

With evolutionary anthropologists increasingly working in (post‐)industrialized contexts, finding a solution to the barriers of naturalistic observations in private worlds seems critical. As one potential solution, we present and reflect on *autophotography*, a participant‐led visual data collection, as a quasi‐naturalistic observational method which may be more feasible in certain population contexts.

## 3 Can We Observe Participants Without Being There? Introducing Autophotography

Across many societies, much of everyday life takes place in private or age‑and gender‑segregated spaces, making focal follows difficult or impossible. Even when researchers get access to private spaces, our presence may be so “unnatural” that it risks amplifying *observer effects* (where participants change their behavior because they are being observed). Within Evolutionary Anthropology, the typical solution to this problem has been to collect behavioral data via survey or interview. However, pre‐defined questions ‐what to ask, and how to ask them‐ are inherently influenced by our own epistemic and cultural assumptions, which can lead to data collection bias [14]. As noted by Darwin and Tinbergen, the key strength of naturalistic observations is to minimize researcher biases. Thus, we need a novel (as well as affordable and accessible) method of collecting data *within the participant's own environment* while *minimizing researcher influence*.

Autophotography is a method with potential, allowing researchers to collect snapshots of quai‐naturalistic observational data within the participant's own environment without researcher presence ‐ thus allowing access to behaviors in private spaces which may otherwise be out of reach. In its simplest form, autophotography generates research data by asking participants to take photographs of their activities or environments using cameras [15, 16], and more recently, mobile phones (e.g., see [17]). Developed within ethnographic traditions and falling under the umbrella of “visual methods,” autophotography emerged as a way to de‐center the researcher and foreground the participants’ own views and experiences of their environments [15] and may be particularly suited to capturing information related to participant socio‐ecologies including local mobility (where the participants go) and social connections (who the participants engage with). While autophotography is not particularly suited to capturing “live behaviors” (after all, participants will be taking photographs!), many behaviors that can be temporarily paused without too much awkwardness come under the scope of autrophotography—such as mapping out daily routines (e.g., see [17]), what foods are eaten (e.g., see [18]), and locations and spaces used or visited (e.g., see [19, 20]). With careful ethical consideration, research design and relationship‐building, autophotography has also been used to capture “hidden” behaviors such as drug use and antisocial behavior (e.g., see [21]).

While autophotograhy studies have tended towards modest sample sizes of fewer than 50 participants [22], our own experience suggests that the method offers practical benefits in terms of efficiency and achievable sample size—particularly when several researchers contribute to data collection. In our project (see Box 1), with a small team, we found collecting data from approximately 30 participants over the course of a week to be entirely feasible; something that would have been nearly impossible using traditional observational methods that rely on time‐intensive focal follows of individual participants.

Box 1 **An example of applying autophotography in cross‐cultural research.**

**Project: Adolescent Sociality Across Cultures – “In My Life” Project**

Website: https://www.adolescentsociality.com/

Publication: [23] 10.1177/07435584251349497

Project Materials: https://osf.io/gdhz8/overview

**Overview**

Led by two Human Behavioral Ecologists with multidisciplinary collaborators, the project was designed and delivered as an exploratory observational study to characterize the lives of adolescents in Japan and England and the behavioral settings they navigate. The findings were intended to provide a conceptual and empirical foundation for future cross‐national research. Adopting a participant‐centered approach that recognizes adolescents as experts in their own everyday environments, we aimed to generate minimally intrusive, context‐rich accounts of adolescence in both settings. We selected autophotography because it could be implemented consistently across the two national contexts; many adolescents in both Japan and England engage with photography through social media, making the method accessible, low‐burden, and well suited to producing comparable data.

**Method Summary**

For this project, we provided point‐and‐shoot cameras to approximately one hundred 13–15‐year‐olds recruited through schools in urban and rural areas in Japan and England, with data collection running from April 2019 to January 2020. Participants were asked to photograph the important aspects of their lives to assess whether this approach could offer a brief but real‐time view into the private worlds of adolescents.

Pre‐fieldwork, several members of the research team met to document our expectations regarding what adolescents might photograph, as a reflexive exercise intended to surface any researcher assumptions that could bias data collection or shape interpretation. The project was designed as a photography project for students, mirroring the type of activities student engaged in at school. Participants first attended a 1‐h workshop where we: (1) introduced the project, (2) explored photography as a way to capture the important things in their lives, (3) explored the ethics of photography including the importance of subject consent, privacy, risks of identification and publishing rights, and (4) practiced how to take photographs using point‐and‐shoot cameras. Students then had approximately 1 week to photograph their lives, followed by a second 1‐h session where students selected that they wanted to submit to the researchers, and gave titles/provided accompanying text to their chosen photographs. The photographs and the accompanying text were analyzed through template analysis; a structured and iterative form of thematic analysis combining deductive and inductive processes. Further information on the methods can be found in [23].

**Brief Reflections: Methodological Success**

The selective nature of photography together with potential participant performance means autophotography is unlikely to be truly “natural.” Nonetheless, in a context where observing participants in their private spaces are highly unusual and likely to alter behavior (or simply impossible), we felt autophotography may succeed as a “quasi‐natural” method to understand behavioral contexts. Firstly, as anticipated, the method seemed successful in capturing moments from the private world of adolescents, with some photographs taken in settings where the presence of adult would have been inappropriate. For example, one participant from London, England, took a photograph in a park at night with friends (see image below)—a situation which would have been difficult for researchers to access and observe. Second, our actual findings deviated somewhat from our expectations, highlighting autophotography's potential to challenge researcher assumptions. For instance, during our pre‐fieldwork meeting, we predicted that participants would take photographs showcasing status (e.g., expensive trainers) and self‐portraits. However, such images were limited. Clearly, what we assumed to be important for adolescents did not align with their actual priorities. Third, the method offered practical benefits in terms of efficiency and increased sample size. With a few researchers, collecting data from approximately 30 participants over a week was entirely feasible; something that would have been nearly impossible with traditional observations.

**Brief Reflections: Methodological Challenges**

We did not capture specific behaviors; rather, we documented the contexts in which behaviors occurred. On reflection, this stemmed from the project's exploratory design, which encouraged participants to decide freely what to photograph. Since this study, Emmott has conducted a further autophotography project in which participants were specifically requested to document daily routines, resulting in a greater proportion of behavior‑focused images (see [17]). Second, although researchers were absent during photography, observer effects were not entirely eliminated: many participants selected only their “best” photographs and declined to share others, indicating an awareness of being viewed by researchers or peers. Third, while participants generally enjoyed using point‑and‑shoot cameras, this technology did not integrate seamlessly into some adolescents’ routines; several reported forgetting to carry the cameras, which limited participation. In a subsequent study with parents, participants were offered a choice between point‑and‑shoot cameras and their own smartphones, and all opted for smartphones (see [17]). Finally, interpretation was sometimes constrained by limited accompanying text, particularly for artistic or abstract images. We had planned post‑collection feedback sessions to elicit further context, but these were canceled due to COVID‑19 restrictions.

**Tips for Future Autophotography Research as a Quasi‐Natural Observation**

1. Provide structured prompts that specify when, where, or what to photograph. Autophotography is highly sensitive to prompt design. Broad prompts may elicit esthetic or identity‑oriented images, whereas temporally or behaviorally framed prompts (e.g., routines, transitions, recurrent settings) are more likely to generate data suitable for quasi‑naturalistic observation.
2. Pair photographs with complementary data to support interpretation. Because images are selective, curated, and often ambiguous, they require additional contextual information. Brief surveys, interviews, diary methods, or photo‑elicitation discussions can supply the behavioral, temporal, and interpretive detail necessary to treat autophotography as an observational tool rather than a purely expressive one.

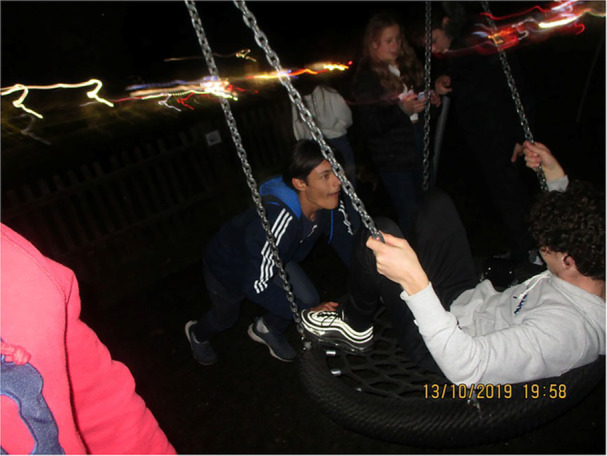

*"late nights at the park” by Orla Hannant‐Clune, 2019 © CC‐BY‐ND*.

At its core, autophotographic methods treat participant‐generated photographs as data. Given its roots in ethnographic research, the photographs are also typically accompanied by additional forms of data such as interviews, photo‐essays and diary methods, providing further context to the images. Photographs and accompanying complementary data are usually analyzed qualitatively, applying methods such as inductive thematic analysis or content analysis—but photographs may also be coded, categorized, an analyzed quantitatively [22]. A case study of our own experience with autophotography exploring the “private worlds” of adolescents in Japan and England is outlined in Box 1.

## 4 Doing Autography Effectively in Evolutionary Anthropology: Knowing the Limitations

A central limitation of autophotography as a quasi‐naturalistic observational method is that participants are typically behind the camera rather than in front of it. This does not preclude the capture of behavior, but it does mean that interpreting behavior often requires researcher inference or participant explanation. For this reason, autophotography may be best complemented with additional forms of data collection, such as written reflections that provide contextual detail (see Box 1 for an example) or photo‑elicitation interviews which can elicit richer accounts ([24]; see [17]). However, these approaches must be designed with care, considering the project aims as well as practical limitations. For example, 1‐2‐1 photo‑elicitation interviews can generate more nuanced descriptions of participants’ lives [25], but they are time‑intensive and may be impractical for studies with large samples.

It is also important to recognize that taking photographs can interfere with everyday life, potentially constraining the “natural behaviors” researchers hope to observe. The impact of the photography must therefore be balanced against the desired granularity of the data. If participants are asked to take images at set intervals, for example, researchers must ensure that this is both feasible and not unduly disruptive. While traditional focal‑follow observations may involve scanning every 90 s, such frequency is unrealistic for participants to replicate. One potential solution is autovideography, which may become increasingly viable with the wider availability of wearable video devices. However, we remain cautious about this approach due to the amplified ethical risks that are discussed below.

Photography is also widely understood as an artistic medium, and this expectation can shape participant behavior. In our project (Box 1), some participants produced abstract or highly stylized images, which was difficult to interpret without additional context. Clear prompts, specifying when, where, or what participants should photograph, may help mitigate this. The choice of photographic tool may also matter. In our experience, asking participants to use their own smartphones produced more natural, incidental images, likely because smartphones are already embedded in the daily routines of many participants. In contrast, providing a separate camera ‐a comparatively novel tool‐ tended to generate more staged photographs. This is not necessarily problematic, but it is an important design consideration.

While past autophotography projects suggest that the absence of researchers during data collection can offer insight into the more “hidden” aspects of participants’ lives (e.g., [21]; see Box 1), observer effects are not fully eliminated. Participants remain aware that their images will be viewed, and studies consistently show that they are selective in what they capture and choose to share. Many autophotography projects explicitly incorporate a stage in which participants select the photographs they wish to share with others (e.g., [19, 23]). This selectivity is not necessarily a limitation depending on the study aims; researchers may intentionally prioritize images that hold significance for participants, rather than striving for an exhaustive visual record. However, if accurate behavioral records are more important than participant‑defined significance, one way to navigate this, perhaps counterintuitively, is to avoid collecting the photographs themselves. This reduces any discomfort or incentive for participants to curate their images. Instead, photographs can be used as a complementary tool to reconstruct participant behaviors through interviews or surveys; for example, researchers and participants might “code” the photographs together during discussion, using the images as prompts rather than as primary data.

Finally, recruitment poses challenges. Autophotography requires sustained participant effort, meaning that samples often rely on self‐selection or convenience recruitment, which can introduce bias [22]. While obtaining a random or fully representative sample is not impossible, a purposive sampling strategy is typically more realistic for this method, particularly when researchers aim to work with participants who are willing and able to engage in participant‑generated visual data collection.

## 5 Navigating Ethical Challenges With Autophotography

A central consideration when undertaking autophotography is the ethical risk inherent in photographic data. Photographs can contain highly sensitive information, including identifiable individuals, private locations, and contextual cues that may be captured without the explicit consent of those depicted. These challenges are well established within anthropology, particularly regarding subject permissions and the ethics of publishing fieldwork images (see, e.g., a useful discussion on the AAA Ethics Forum: https://ethics.americananthro.org/ethical‐considerations‐when‐publishing‐fieldwork‐photos‐in‐online‐sources‐2/).

Ethical concerns extend beyond participants themselves to the subjects of their photographs, making it essential to provide clear guidance to participants on what can and cannot be photographed for the safety of all involved (e.g., see [21]). It is also important to remember that legislation and norms around photography differ between countries. Participant training and ongoing support are therefore fundamental, even when participants are already familiar with photography, as the method asks them to engage with their environments in new ways and, at times, to obtain consent from others. In our own project with adolescents, all participants attended an information session and workshop where we explained the importance of privacy, legal requirements, subject consent. We also practised seeking consent and taking photographs responsibly (see Box 1).

If photographs are intended for public dissemination (including as open data), when subjects are identifiable or when images raise ethical concerns, the photographs may require editing, or some images may not be publishable. These potential processes must be communicated transparently during the onboarding and consent process, before data collection. Finally, issues of copyright and ownership must be addressed. When participants retain ownership of their images, such as under a CC‐BY licence, they also retain responsibility for any associated risks, and this should be clearly acknowledged within the consent process.

## 6 Final Thought: Autophotography in Evolutionary Anthropology

Naturalistic observation has long been central to evolutionary anthropology, yet its use has declined as it has become increasingly impractical in many contemporary settings. Despite its limitations and challenges, autophotography may help recover some of the strengths of naturalistic observation while adapting to the constraints of modern field sites, allowing us to collect “real‐time” data without direct researcher presence. Whether used in an exploratory or confirmatory manner, autophotography may reveal unexpected insights by allowing a direct view into the lives of our participants within their own communities and environments, which is the key strength of the method.

Autophotogrpahy will not be suitable for every project, and our intention is not to recommend this method as superior to other approaches such as behavioral surveys or experiments methods. Rather, we present autophotography as a useful addition to the methodological toolkit of evolutionary anthropologists given our pre‐existing knowledge and understanding of participant observations. For example, when embarking on new projects with new communities, autophotography may help researchers to develop insight into local norms and environments alongside specific participant behaviors. Such contextual knowledge can strengthen study design, support the development of culturally appropriate methods, and enhance the interpretation of behavioral findings.

## Ethics Statement

The authors have nothing to report.

## Consent

The authors have nothing to report.

## Conflicts of Interest

The authors declare no conflicts of interest.

## Data Availability

Data sharing not applicable to this article as no datasets were generated or analyzed during the current study. Published photographs can be accessed at https://www.adolescentsociality.com/photos. Further information on our methods and project details can be found in Emmott et al. (2025), https://osf.io/gdhz8/, and 10.33424/FUTURUM204. This paper is a revised version of a preprint in *SocArXiv*: 10.31235/osf.io/q87hu_v1.
